# Diagnosis of Syphilis

**Published:** 1903-04-04

**Authors:** 


### DIAGNOSIS OF SYPHILIS.

In a paper published in the Brooklyn *Medical Journal* this month Dr. Muren discusses fully the difficulty, and in many instances the complete impossibility, of making a correct diagnosis of primary syphilis, and he urges, with some force, that both the diagnosis and the treatment by mercury in every suspected case ought be deferred until the nature of the disease has been made clear by the appearance of some general manifestations of the disease. He states that he can find but one American author who believes in the early use of mercury, that is to say, before the appearance of "secondaries." The reasons which Dr. Muren gives for this procrastination are, in the first place, the extreme seriousness of making a mistake in the diagnosis when the questions of marriage, paternity, and the patient's general mode of life will be gravely affected. In the second place, if well-marked secondary manifestations occur, the patient is more likely to be convinced that he has really been infected with syphilis, and will, for this reason, be more likely to submit to a prolonged course of treatment. Lastly, it is held that no harm will result from the delay, and that the early mercurial treatment, which is in general use in other countries, does not materially affect the subsequent course of the disease. The advocates of the early administration of mercury, on the other hand, claim that when it is used promptly the subsequent course of the disease is less severe, and that this is shown by the generally admitted fact that the secondary manifestations are considerably modified, if not altogether prevented in some instances, under an early applied mercury regime.

